# Different Functions of the Insect Soluble and Membrane-Bound Trehalase Genes in Chitin Biosynthesis Revealed by RNA Interference

**DOI:** 10.1371/journal.pone.0010133

**Published:** 2010-04-12

**Authors:** Jie Chen, Bin Tang, Hongxin Chen, Qiong Yao, Xiaofeng Huang, Jing Chen, Daowei Zhang, Wenqing Zhang

**Affiliations:** 1 State Key Laboratory of Biocontrol, School of Life Sciences, Sun Yat-sen University, Guangzhou, China; 2 Hangzhou Key Laboratory of Animal Adaptation and Evolution, Hangzhou Normal University, Hangzhou, Zhejiang, China; University Paris Diderot-Paris 7, France

## Abstract

**Background:**

Trehalase, an enzyme that hydrolyzes trehalose to yield two glucose molecules, plays a pivotal role in various physiological processes. In recent years, trehalase proteins have been purified from several insect species and are divided into soluble (Tre-1) and membrane-bound (Tre-2) trehalases. However, no functions of the two trehalases in chitin biosynthesis in insects have yet been reported.

**Principal Findings:**

The membrane-bound trehalase of *Spodoptera exigua* (*SeTre-2*) was characterized in our laboratory previously. In this study, we cloned the soluble trehalase gene (*SeTre-1*) and investigated the tissue distribution and developmental expression pattern of the two trehalase genes. *SeTre-1* was expressed highly in cuticle and Malpighian tubules, while *SeTre-2* was expressed in tracheae and fat body. In the midgut, the two trehalase genes were expressed in different locations. Additionally, the expression profiles of both trehalase mRNAs and their enzyme activities suggest that they may play different roles in chitin biosynthesis. The RNA interference (RNAi) of either *SeTre-1* or *SeTre-2* was gene-specific and effective, with efficiency rates up to 83% at 72 h post injection. After RNAi of *SeTre-1* and *SeTre-2*, significant higher mortality rates were observed during the larva-pupa stage and pupa-adult stage, and the lethal phenotypes were classified and analyzed. Additionally, the change trends of concentration of trehalose and glucose appeared reciprocally in RNAi-mutants. Moreover, knockdown of *SeTre-1* gene largely inhibited the expression of chitin synthase gene A (*CHSA*) and reduced the chitin content in the cuticle to two-thirds relative to the control insects. The chitin synthase gene B (*CHSB*) expression, however, was inhibited more by the injection of dsRNA for *SeTre-2,* and the chitin content in the midgut decreased by about 25%.

**Conclusions:**

*SeTre-1* plays a major role in *CHSA* expression and chitin synthesis in the cuticle, and *SeTre-2* has an important role in *CHSB* expression and chitin synthesis in the midgut.

## Introduction

Trehalase (α-glucoside-1-glucohydrolase, EC 3.2.1.28) is an enzyme that hydrolyzes trehalose to yield two glucose molecules, and it is present in almost all tissues in different forms. Trehalase plays a pivotal role in various physiological processes, including flight metabolism[1], chitin synthesis during molting [2], and cold tolerance[3]. In insects, all these function of trehalase are achieved through the hydrolysis of trehalose (α-D-glucopyranosyl-α-D-glucopyranoside), the principal hemolymph sugar in insects that acts as an indispensable substrate for energy production and macromolecular biosynthesis[4].

The chitin biosynthesis pathway starts with trehalose[5], and the first enzyme involved in the pathway is trehalase, and the last one is chitin synthase. Other enzymes involved include hexokinase, Glucose-6-P isomerase (G-6-P-I), UDP-N-acetylglucosamine pyrophosphorylase (UAP) and so on. The activity of enzymes involved in chitin metabolism were affected by temperature, especially at elevated temperature[6]. To date, trehalase proteins have been purified from various insect species and are divided into the soluble (Tre-1) and the membrane-bound (Tre-2) trehalases [7], [8], [9], [10], [11], [12]. The first insect trehalase gene, a soluble trehalase gene, was reported in 1992[13], and a membrane-bound trehalase gene was not reported until 2005[14].

The number of genes encoding a family of proteins in insects varies widely among species. For example, two trehalase genes, two chitin synthase genes, and 13 to 16 chitinase-related genes have been identified thus far in insects[15], [16], [17]. Different genes in a family often have different functions. The chitin synthase gene A of *Tribolium castaneum* (*TcCHSA*) is the sole contributor to cuticular chitin synthesis, and chitin synthase gene B (*TcCHSB*) is the major/sole contributor to the chitin synthesis in peritrophic matrix chitin synthesis[18]. The chitinase genes in *Tribolium castaneum* differ in their expression patterns during development and in different tissues, and genes in different groups have different functions[19]. In some situations, isoforms of a gene are functionally redundant[20]. However, the functions of the two trehalases in insect chitin biosynthesis have not yet been reported.

The beet armyworm *Spodoptera exigua* is a serious lepidopteran pest. Its membrane-bound trehalase gene was previously characterized in our laboratory[16]. Here we cloned the soluble trehalase gene. Different expression pattern of the two trehalase genes suggest that they may have different functions. The transcript levels of the two genes were substantially down-regulated by injection of gene-specific dsRNAs of the two trehalases. The results above revealed that the two genes have different functions in insect chitin biosynthesis.

## Results

### Cloning and sequence analysis of *SeTre-1* cDNA

The full-length cDNA sequence of the soluble trehalase in *S. exigua* (*SeTre-1*) is 2144 nucleotides with an open reading frame of 1755 nucleotides, which encodes a protein of 585 amino acids with a predicted mass of approximately 66.48 KDa and an isoelectric point of 4.75.**** The deduced amino acid sequence of *SeTre-1* was aligned with the trehalases of other insect species. Compared with the membrane-bound trehalase gene (*SeTreh-2*)[16], they have only 37% homeology.

**Figure 1:**
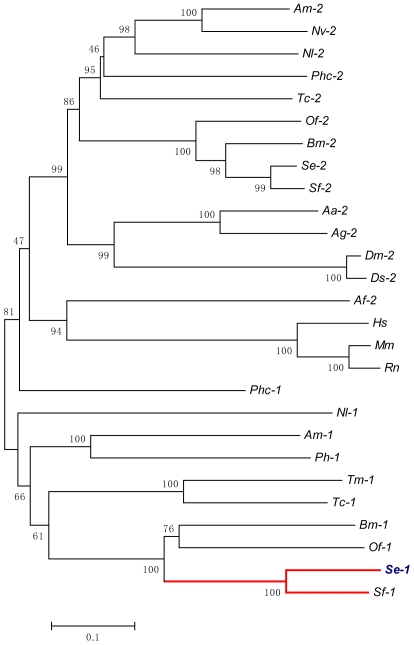
Phylogenetic analysis of the trehalase amino acid sequences. The phylogenetic analysis of the trehalases amino acids was conducted using the MEGA3.1 program to generate a phylogenetic tree (1, the soluble trehalase gene; 2, the membrane-bound trehalase gene) and the robustness of each cluster was verified in 1000 replicates. The scale on the x-axis represents the estimated branch lengths and numbers indicate the bootstrap values. Trehalases were from *Aedes aegypti* (Aa), *Anopheles gambiae*(Ag), *Apis mellifera* (Am), *Bombyx mori* (Bm), *Drosophila melanogaster* (Dm), *Drosophila simulans* (Ds), *Nasonia vitripennis* (Nv), *Ostrinia furnacalis* (Of), *Pimpla hypochondriaca* (Ph), *Spodoptera exigua* (Se), *Spodoptera frugiperda* (Sf), *Tribolium castaneum* (Tc), *Nilaparvata lugensand* (Nl), *Tenebrio molitor* (Tm), *Mus musculus* (Mm) and *Homo sapiens* (Hs). The GenBank numbers (cDNA) are as follows: *Aa-2* (XM_001660243), *Ag-2* (XM_320471), *Am-2* (NM_001112671), *Bm-2* (NM_001043445), *Dm-2* (DQ864060), *Ds-2* (DQ864075), *Nv-2* (XM_001602129), *Of-2* (EF426723), *Tc-2* (XM_967517), *Nl-2* (GQ397451), *Sf-2* (EU872435), *Se-2* (EU106080), *Bm-1* (D86212), *Of-1* (EF426724), *Ph-1* (AJ459958), *Sf-1* (DQ447188), *Se-1* (EU427311), *Tc-1* (XM_968826), *Tm-1* (D11338), *Am-1* (XM_393963), *Nl-1* (EJ790319), *Mm* (NM_021481), *Hs* (NM_007180).

A phylogenic tree was constructed based on the full-length sequences of known trehalases from insects and other organisms (Figure 1). The results showed that the overall amino acid sequence of *SeTre-1* shares high identity with *Spodoptera frugiperda Tre-1* (80%) (DQ447188), *Ostrinia furnacalis Tre-1* (55%) (EF426724), *Bombyx mori Tre-1* (55%) (D86212), followed by *Tribolium castaneum Tre-1* (44%) (XM_968826), *Nilaparvata lugens Tre-1* (41%) (EJ790319), *S. exigua Tre-2* (37%) (EU106080), *S. frugiperda Tre-2* (36%) (EU872435), *B. mori Tre-2* (36%) (NM_001043445), *N. lugens Tre-2* (36%) (GQ397451), and *T. castaneum Tre-2* (35%) (XM_967517).

### Immuno-blotting in tissues

**Figure 2:**
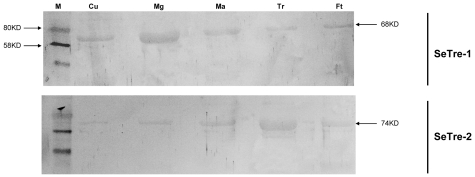
Immuno-blot analysis of SeTre-1 and SeTre-2 in various tissues. 10 ug protein extracts from the crude tissue extracts were loaded on 12 % SDS-PAGE. Lanes were immuno stained with anti-SeTre-1 serum or anti-SeTre-2 serum. Arrows indicated the positions of molecular weight markers. Cu: cuticle; Mg: midgut; Ma: Malpighian tubules; Tr: tracheae; Ft: fat body.

Immuno-blot analysis was performed with anti-SeTre-1 or anti-SeTre-2 serums. Only one major protein at 68 or 74 kDa was detected in the tissues on day 2 of fifth instars (Figure 2). The size was consistent with the predicted mass of SeTre-1 or SeTre-2 deduced from amino acid sequence. These data suggested that the polyclonal antibodies specifically recognized SeTre-1 or SeTre-2.

### Immunocytochemical analysis of SeTre-1 and SeTre-2

**Figure 3:**
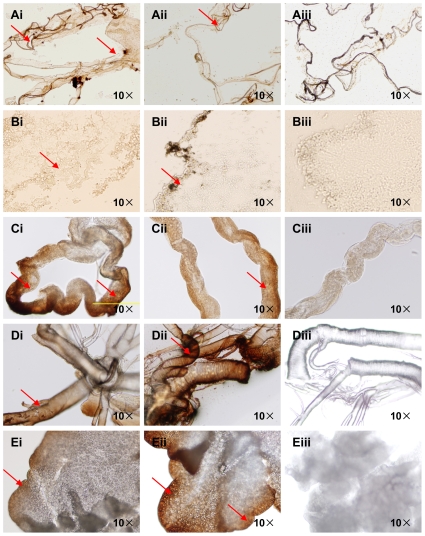
Immunocytochemical analysis of SeTre-1 and SeTre-2 in various tissues. Various tissues were dissected on day two of the fifith instar larvae and incubated with anti-SeTre-1 or 2 sera followed by goat anti-rabbit IgG conjugated with DAB. Cuticle (A), midgut (B), Malpighian tubules (C), tracheae (D) and fat body (E) were stained (red arrow heads indicate the positive staining). Tissues immunostained with anti-SeTre-1 (Ai-Ei) serum and anti-SeTre-2 (Aii-Eii) serum were stained respectively. Cryosections and tissues immunostained with preimmune serum were used as negative controls (Aiii-Eiii).

The localization of SeTre-1 and SeTre-2 proteins in the midgut, cuticle, tracheae, Malpighian tubules and fat body were determined by immunohistochemistry. Different expression levels of SeTre-1 and SeTre-2 were detected in all tissues (Figure 3). *SeTre-1* was highly expressed in the cuticle, middle section of the midgut and Malpighian tubules but weakly in the fat body, while *SeTre-2* was expressed in the verge section of the midgut, and highly expressed in the tracheae and fat body but very weakly in the cuticle. Additionally, the expressions of *SeTre-1* and *SeTre-2* mRNA in different tissues were also determined by semi-quantitative RT-PCR, coincident results were obtained (data not shown).

### Developmental expression pattern of *SeTreh-1* and *SeTre-2*

**Figure 4:**
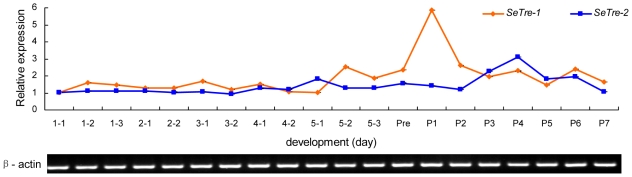
Developmental profiles of Se*Tre-1* and Se*Tre-2* mRNA expression during the larva-pupa-adult metamorphosis. The levels of both *SeTre-1* and *SeTre-2* mRNAs relative to the *Seβ-actin* mRNA level were measured with real-time PCR. Each point represents the mean ± SEM from three independent experiments. The age of the insects is indicated in days: 1-1, the first day of the first instar larva; P1, the first day of the pupal stage.

The expression patterns of the two trehalases during *S. exigua* development from day one of the first instar larva through pupa to adult were determined by quantitative real-time PCR. As the mRNA levels of *Seβ-actin* gene were nearly constant during the whole development stages (Figure 4), it was used to standardize the relative expression levels of *SeTre-1* and *SeTre-2* genes.[21] *SeTre-1* and *SeTre-2* were continuously expressed in all developmental stages. After day one of the fifth instar, the *SeTre-1* mRNA expression increased gradually and reached a maximal level at day one of the pupae, while the mRNA level of *SeTre-2* remained at a constant level and increased to a high level at day four of the pupae (Figure 4). The different temporal expression patterns suggested distinct physiological roles of the two trehalases.

### Enzyme activity of the two trehalases

In addition, enzyme activities of SeTre-1 and SeTre-2 were determined at the three important metamorphosis stages, from the fourth larva to fifth larva, from larva to pupa and from pupa to adult. In the haemolymph, the SeTre-1/SeTre-2 ratio remained almost constant, reflecting a dynamic balance of the insect blood sugar, but the total activity of SeTre-1 and SeTre-2 in individual insects changed dramatically at various stages. For example, SeTre-2 activity was about two-fold higher than that of SeTre-1 at the late fourth instar stage, while SeTre-1 activity was about three-fold and about 1.5-fold higher than SeTre-2 in the prepupal stage and late pupal stage, respectively (Table 1). These results suggest that SeTre-1 plays a crucial role in the larva-pupa and pupa-adult stages, and SeTre-2 contributes more in the pupa-adult stage.

**Table 1:** Enzyme activity of SeTre-1 and SeTre-2 during the fourth larva to the fifith larva, larva-pupa and pupa-adult stages in *S. exigua*.

	type	End of 4^th^ instar larva (nmol glucose/ug protein/min)	Prepupa (nmol glucose/ug protein/min)	7^th^ day of pupa (nmol glucose/ug protein/min)
hemolymph	SeTre-1	7.53±1.42	5.66±0.60	4.45±0.62
	SeTre-2	1.96±0.64	1.34±0.62	1.83±0.71
individual	SeTre-1	16.32±2.29	103.9±0.83	22.95±0.61
	SeTre-2	29.14±1.10	36.90±2.05	14.35±0.59

Each data point is the mean ± SEM of three independent experiments with ten individuals each (n = 30).

### Efficiency and specificity of RNAi for *SeTre-1* and *SeTre-2*

**Figure 5:**
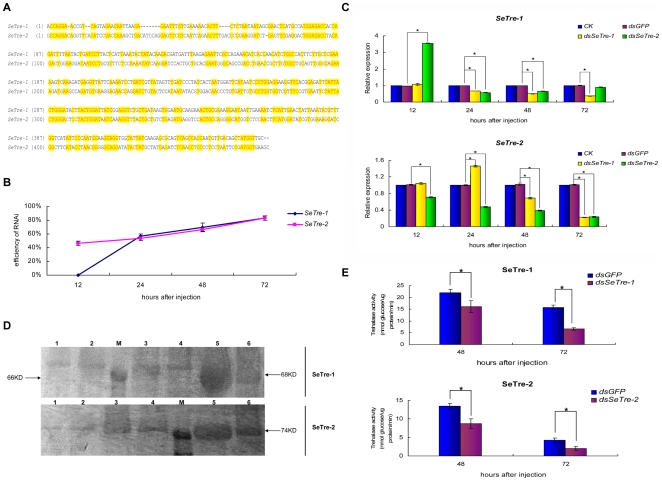
Changes in the mRNA, protein and enzyme activity levels of *SeTre-1* and *SeTre-2* after specific RNAi. (A) An alignment of the nucleotide sequences of *SeTre-1* and *SeTre-2* in the region of the dsRNAs. (B) The efficiency of RNAi for *SeTre-1* and *SeTre-2*. Each point represents the mean ± SEM from three independent experiments. (C) The transcription levels of target genes and the other trehalase gene were detected. Both the mRNA levels of *SeTre-1* and *SeTre-2* relative to the *Seβ-actin* mRNA level were measured with real-time PCR. The mRNA expression level in the non-injection group is designated as one. Each point represents the mean ± SEM from three independent experiments with all effective individuals for target gene detection and with three effective individuals for the other trehalase gene detection. An asterisk indicates significant differences of the mRNA levels between the ds*GFP* and ds*SeTre-1* or ds*SeTre-2* groups measured at the same time (p<0.05, T test). (D) The protein levels of target genes were detected by Western blot. A total of 30 ug of proteins from each individual treated with ds*GFP* or dsRNA was applied to each lane and separated by 12% SDS-polyacrylamide gel electrophoresis. Lanes 1, 3 and 5: 24, 48 and 72 hours after ds*GFP* injection, respectively; Lanes 2, 4 and 6: 24, 48 and 72 hours after ds*SeTre-1* or *2* injection, respectively. (E) SeTre-1 and SeTre-2 activities were measured after dsRNAs injection. Each point represents the mean ± SEM from three independent experiments with three effective individuals in each replicate. Asterisks indicate significant differences of the activities between the two groups measured at the same time (p<0.05, T test).

To verify the specificity of RNAi for *SeTre-1* and *SeTre-2* genes, the ds*SeTre-1* fragment (460 bp) was aligned with the ds*SeTre-2* fragment (475 bp), and 19-bp consecutive identical sequences between the two fragments were not found (Figure 5A). Because *SeTre-1* was expressed at a higher level in the prepupal stage, whereas *SeTre-2* had high expression level at day one of the fifth instar larvae (Figure 4), the dsRNAs for *SeTre-1* or *SeTre-2* were injected at early day one of the fifth instar stage, prior to the anticipated time of high expression of the two trehalase genes, in order to achieve the maximum effect of down-regulation. Quantitative real-time PCR was carried out using total RNA extracted from dsRNA-injected insects as templates. According to three independent experiments, the efficiency rates of RNAi for both *SeTre-1* and *SeTre-2* increased gradually and reached 83% at 72 hours post-injection (An individual with more than a 10% of decrease of target gene expression was regarded as displaying an effective RNAi response) (Figure 5B). The transcript levels of the target genes were compared to those of the controls(non-injection), but decrease in the *SeTre-1* expression level was less than that of *SeTre-2* transcript for equal amounts of dsRNAs, with a 60% decrease in *SeTre-1* gene expression and an 80% decrease in *SeTre-2* gene expression after 72 hours (Figure 5C). Moreover, the protein level changes of the target genes showed that both SeTre-1 and SeTre-2 decreased immune signals in response to the dsRNAs injection from 48 hours post-injection, especially at 72 hours (Figure 5D). Enzyme activities of both trehalaes also presented significant decrease from 48 hours, which substantiates the success of the RNAi (Figure 5E).

As shown in figure 5C, after RNAi of *SeTre-1* gene, the *SeTre-2* expression did not decrease even though *SeTre-1* expression dropped obviously at 24 hours post-injection, and after RNAi of *SeTre-2,* the *SeTre-1* expression increased while *SeTre-2* expression reduced markedly at 12 hours post-injection, which demonstrated gene specific RNAi of the either gene in the early period of post-injection. On the other hand, although significant decrease of *SeTre-2* expression from 48 to 72 hours in response to ds*SeTre-1* injection and of *SeTre-1* in response to ds*SeTre-2* injection at 48 hours was detected, it was due to feedback mechanism of the two trehalases (Figure 5C). These results indicated the dsRNA-mediated silencing of trehalase was gene-specific within at least 24 hours post-injection.

### Phenotype analysis after RNAi

**Figure 6:**
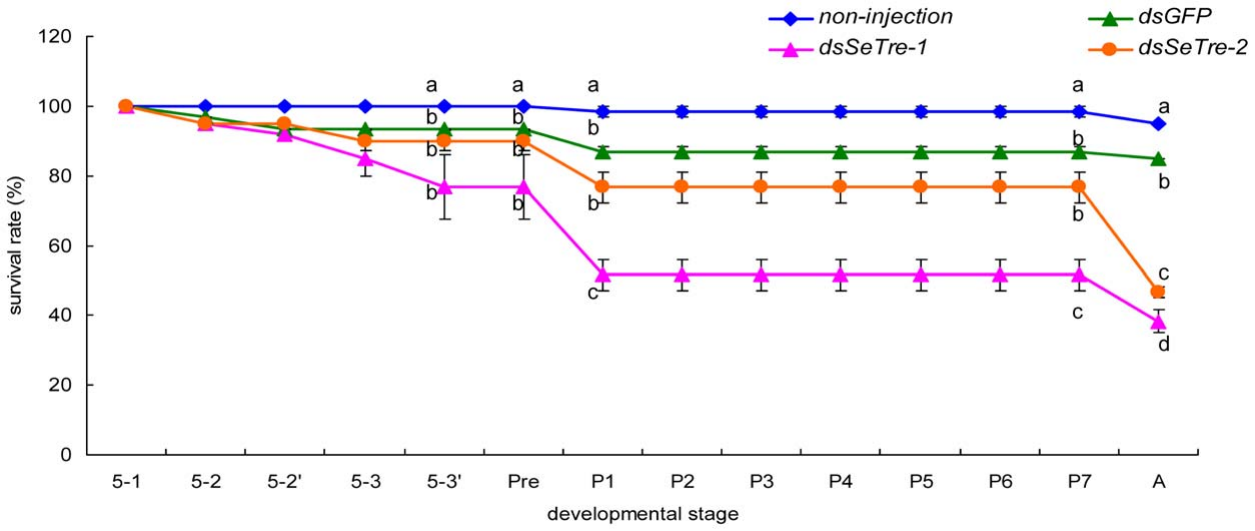
Survival rates after injection of dsRNA of *SeTre-1* and *SeTre-2*. The survival rate of insects after dsRNA of *SeTre-1* and *2* injections during the fifith instar to adult stage. Each point represents 12 hours after the injection before the pupa stage. The age of insects is indicated in days: 5–1, the first day of the fifth instar larva; 5–2 and 5–2′ represent the two 12 hours in one day; P1, the first day of the pupal stage. Each point represents the mean ± SEM from three independent experiments with forty individuals in each group. Different letters indicate significant differences of the survival rates (p<0.05, LSR, SPSS).

After the successfully gene silencing of *SeTre-1* and *SeTre-2*, we investigated whether the inhibition of mRNA expression of the two trehalase genes leads to abnormal or lethal phenotypes. During the larva-pupa stage, 48% and 23% of individual insects injected with dsRNA for *SeTre-1* and *SeTre-2*, respectively, died before the pupal stage, ang these were significantly higher than the mortality rates of the naive insects or insects injected with dsRNA for *GFP*(Figure 6). During the pupa-adult stage, 13% and 30% of individual insects injected with dsRNA for *SeTre-1* and *SeTre-2*, respectively, exhibited the malformation phenotype, and these were significantly higher than the two control groups (Figure 6).

**Figure 7:**
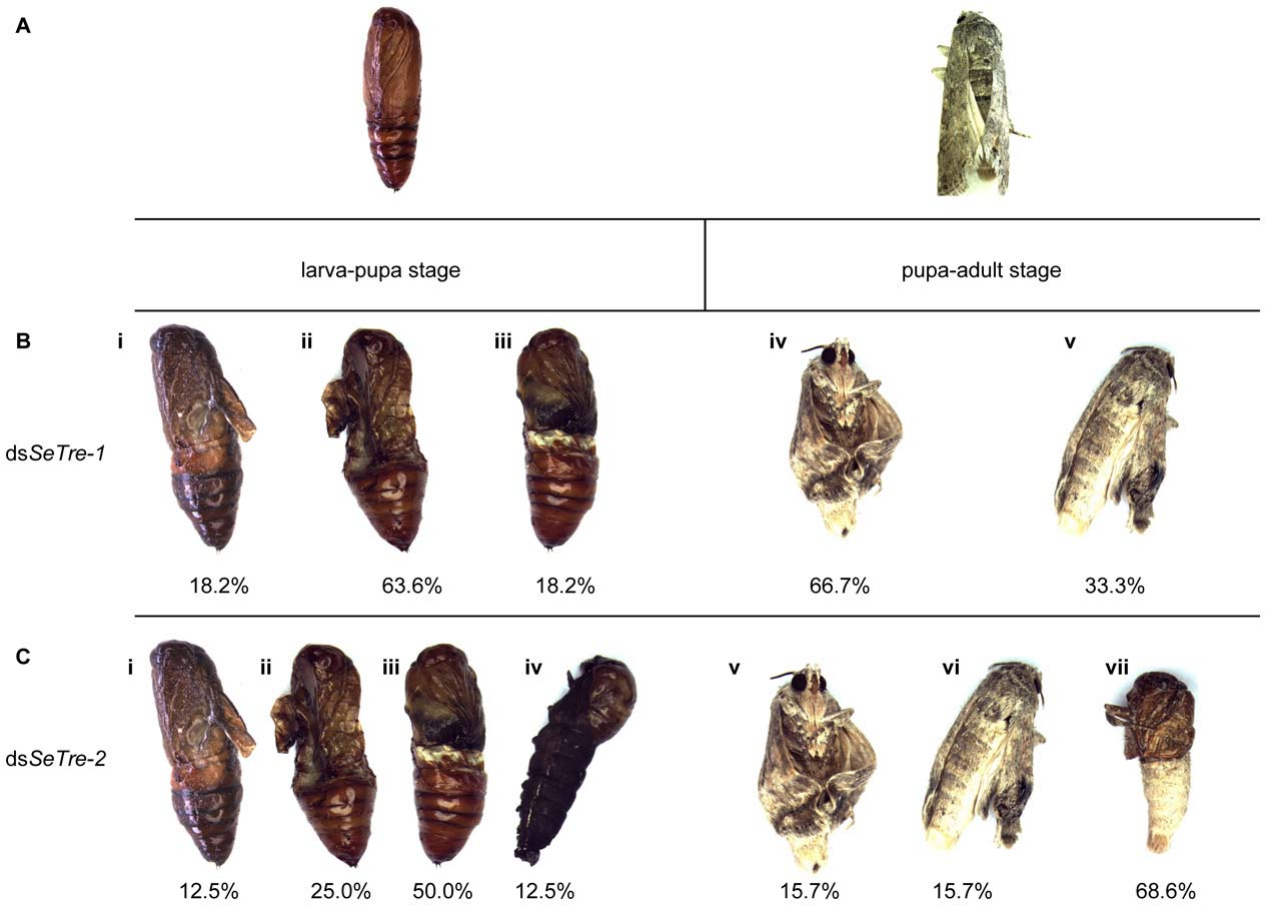
Lethal phenotypes caused by the RNAi for *SeTre-1* or *SeTre-2*. During the larva-pupa stage, the insects injected with dsRNA of *SeTre-1* produced three different lethal phenotypes, with a 63.6% proportion of the “severe-abnormal” phenotype (Bi-iii), while the insects injected with dsRNA of *SeTre-2* produced four different lethal phenotypes, with a 50% proportion of the “abdomen-abnormal” phenotype during the larva-pupa stage (Ci-iv). During the pupa-adult stage, the insects injected with dsRNA of *SeTre-1* produced two different lethal phenotypes, with a 66.7% proportion of the “misshapen-wings” phenotype (Biv-v), while the insects injected with dsRNA of *SeTre-2* produced three different lethal phenotypes, with a 68.6% proportion of the “half-eclosion” phenotype (Cv-vii). The pupae and adults in the ds*GFP-*injected control are shown in A.

More specifically, during the prepupa-pupa metamorphosis, 27% and 13% of individuals (about 32 and 16 individuals in three experiments, respectively) injected with dsRNAs for *SeTre-1* and *SeTre-2* exhibited an obviously abnormal phenotype, respectively (Figure 6). Three different lethal phenotypes were observed after the knockdown of *SeTre-1* (Figure 7Bi-iii), with 64% of the malformed individuals exhibiting the “severe-abnormal” phenotype overall, while four different lethal phenotypes were observed after knockdown of *SeTre-2* (Figure 7Ci-iv), with 50% of the malformed individuals exhibiting the “abdomen-abnormal” phenotype. In addition, after 24–48 hours, many individuals injected with dsRNA for *SeTre-2* shrank in body size and had a reduced food intake (data not shown). During the pupa-adult metamorphosis, 13% of the individuals (about 16 total in three experiments) injected with dsRNA for *SeTre-1* displayed an abnormal phenotype, while 30% of the individuals (about 36 total in three experiments) injected with dsRNA for *SeTre-2* displayed an abnormal phenotype (Figure 6). Two eclosion malformation phenotypes were observed with dsRNA for *SeTre-1* (Figure 7Biv-v), and 67% individuals were still alive with misshapen wings (Figure 7Biv). Three eclosion malformation phenotypes were observed with dsRNA for *SeTre-2* (Figure 7Cv-vii), and 69% individuals died without any splitting of the old cuticle (Figure 7Cvii).

A correlation analysis showed that a positive correlation between the efficiency rates of RNAi for *SeTre-1* or *SeTre-2* and the mortality rates 12 h to 72 h post-injection (Pearson correlation coefficient is equal to 0.957* for *SeTre-1* and 0.930 for *SeTre-2*, * means the correlation is significant, SPSS). This result confirmed that malformation phenotypes were caused by accumulative decrease in transcription levels of the two trehalases.

### Changes of trehalose and glucose concentration after RNAi

**Figure 8:**
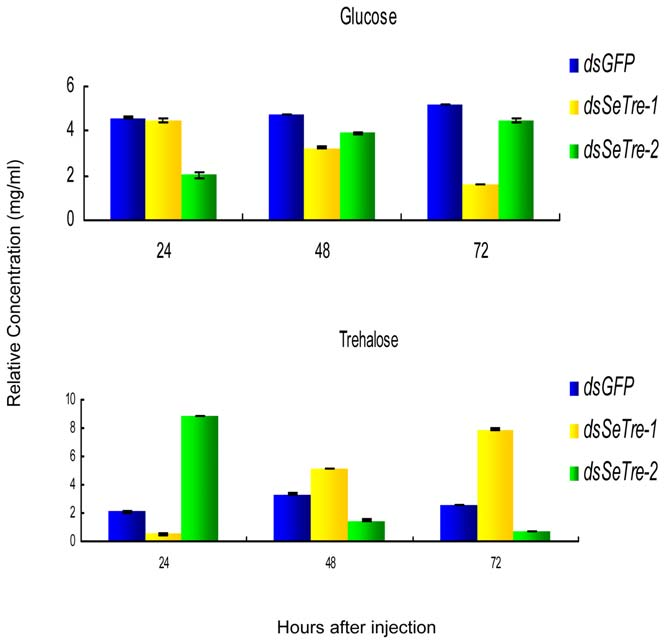
Concentration changes of trehalose and glucose. The concentrations of trehalose and glucose were detected by HPLC. Each point represents the mean ± SEM from three independent experiments with five abnormal individuals mixed in each replicate.

From 24 to 72 hours after the injection of *dsSeTre-1*, the trehalose level increased while the glucose level dropped gradually, but the glucose level increased while the trehalose level decreased after the injection of *dsSeTre-2* (Figure 8). As already shown, after RNAi for *SeTre-1*, the expression level of *SeTre-2* increased initially followed by a persistent reduction until the prepupal stage, leading to a severe shortage of glucose in the pupal progress, which may cause pupation malformation (Figure 5C, Figure 8). On the other hand, after the injection of dsRNA for *SeTre-2,* the *SeTre-1* expression level increased rapidly at the beginning (Figure 5C), while persistent RNAi for *SeTre-2* inhibited the trehalose hydrolyzation from external food-intake, and the transcriptional level of *SeTre-1* dropped significantly, leading to an abundance of trehalose and a shortage of glucose at 24 hours (Figure 5C, Figure 8). After 24 hours, in order to complete metamorphosis and maintain normal activity, trehalose was excessively hydrolyzed into glucose, resulting in enough glucose for the larva-pupa stage. However, this resulted in a severe shortage of trehalose in the pupal stage and may have caused eclosion malformation (Figure 5C, Figure 8).

### Effects of *SeTre-1* and *SeTre-2* RNAi on the expressions of key genes in the chitin biosynthesis pathway

**Figure 9:**
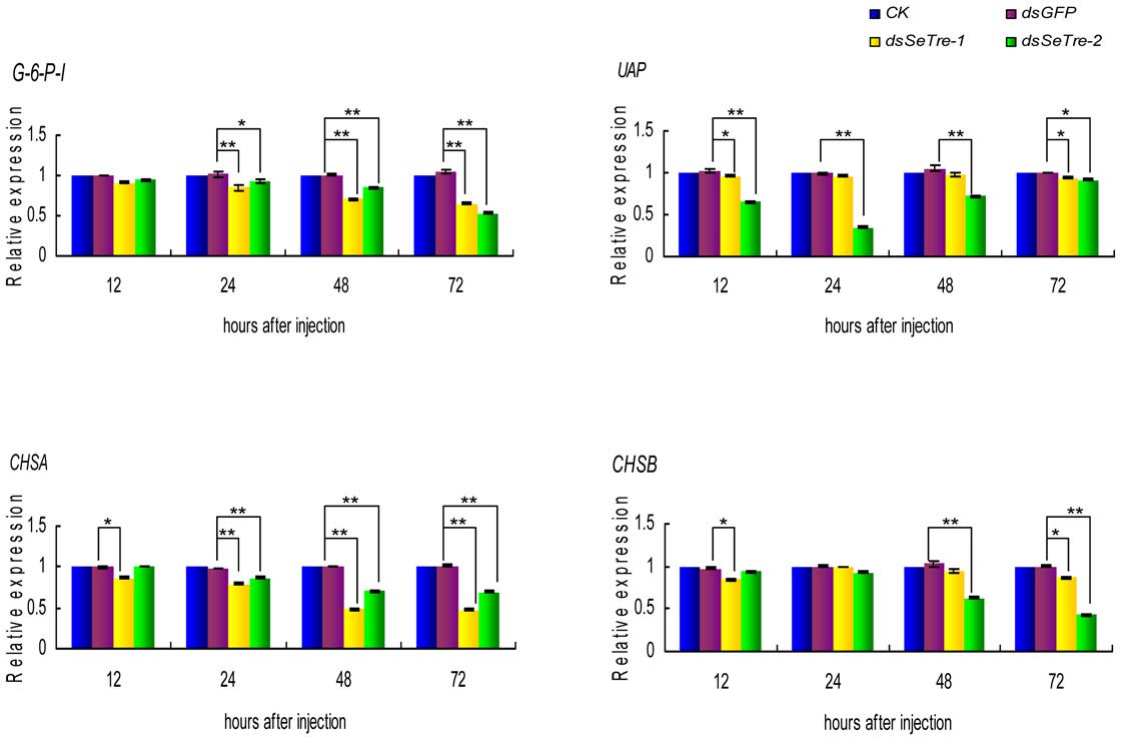
Effects of *SeTre-1* or *SeTre-2* RNAi on key genes in the chitin biosynthesis pathway. The transcription levels of four pathway genes were detected. The levels of both mRNAs relative to the *Seβ-actin* mRNA level were measured with real-time PCR. The mRNA expression level in the non-injection group is designated as one. Each point represents the mean ± SEM from three independent experiments with three effective individuals in each replicate. An asterisk indicates significant differences of the mRNA levels between the ds*GFP* and ds*SeTre-1* or ds*SeTre-2* groups measured at the same time (p<0.05, T test) and double asterisks indicates very significant differences (p<0.01, T test). *G-6-P-I*: glucose-6-phosphate isomerase gene, *UAP*: UDP-N-acetylglucosamine pyrophosphorylase gene, *CHSA*: chitin synthase gene A, *CHSB*: chitin synthase gene B.

Quantitative real-time PCR was used to detect the mRNA levels of four genes in the chitin biosynthesis pathway after the RNAi knockdown of *SeTre-1* and *SeTre-2*. The results showed that *G-6-P-I* (glucose-6-phosphate isomerase) gene was down-regulated extremely significantly by the the RNAi of *SeTre-1* from 24 hours and by the RNAi of *SeTre-2* from 48 hours, while *UAP* (UDP-N-acetylglucosamine pyrophosphorylase) gene expression was affected obviously only by the RNAi of *SeTre-2* from 12 to 48 hours (Figure 9). Moreover, knockdown of *SeTre-1* affected the expression of *CHSA* (chitin synthase gene A) more significantly than *CHSB* (chitin synthase gene B), while the RNAi of *SeTre-2* affected *CHSB* expression more significantly (Figure 9).

### Chitin content in cuticle and midgut after RNAi

The significant decrease in the *CHSA* and *CHSB* transcript levels and the abnormal phenotypes of insects after RNAi of *SeTre-1/SeTre-2* suggest that the chitin content in the insect cuticle and midgut may be decreased. To confirm this hypothesis, the chitin contents in the cuticle and midgut of dsRNA-injected insects were determined at 60 hours post-injection. Compared to the control group, the chitin content in the cuticle of insects injected with dsRNA for *SeTre-1* was reduced greatly, and the mean chitin content per insect was only about two thirds of that in the control insects. In contrast, the chitin content in the insects injected with dsRNA for *SeTre-2* was only slightly reduced. However, compared to the chitin content in the midgut of the control insects, the chitin content in the midgut of the insects injected with dsRNA for *SeTre-2* was reduced about 25%, while the chitin content in the insects injected with the dsRNA of *SeTre-1* did not decrease significantly (Table 2).

**Table 2:** Effects of the injection of Se*Tre-1* or Se*Tre-2* dsRNA on the chitin content in epidermis (Ep) and midgut (Mg) of *S. exigua* larvae.

	Group	Content (ug chitin/*S. exigua*)
Ep	ds*GFP*	65.15±0.50 a
	ds*SeTre-1*	44.56±1.90 b
	ds*SeTre-2*	57.12±1.04 c
Mg	ds*GFP*	2.04±0.90 a
	ds*SeTre-1*	1.99±0.10 a
	ds*SeTre-2*	1.42±0.07 b

Each data point is the mean ± SEM of three independent experiments with ten abnormal individuals each (n = 30). Different letters indicate significant differences of the chitin content (p<0.05, LSR, SPSS).

### Effects of trehalose injection on expressions of key genes in the chitin biosynthesis pathway

**Figure 10:**
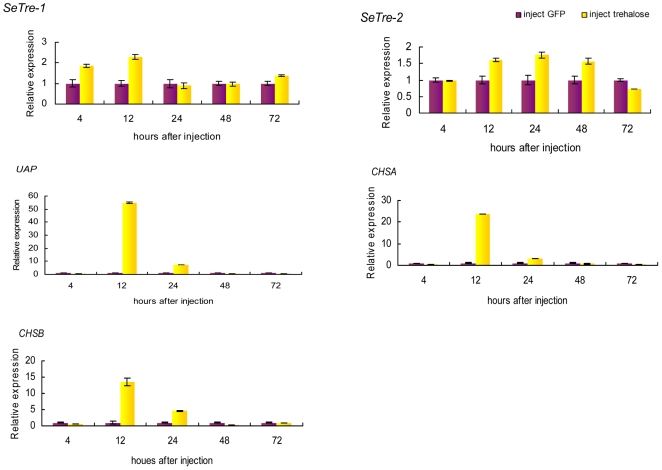
Effects of trehalose injection on expression of the chitin biosynthesis pathway genes, *SeTre-1,* *SeTre-2,* *UAP,* *CHSA* and *CHSB*. The transcription levels of the five genes were detected after injection with a 500 ug dose. Both of the mRNA levels relative to the *Seβ-actin* mRNA level were measured with real-time PCR. The mRNA expression level in the ds*GFP*-injection group is designated as one. Each point represents the mean ± SEM from three independent experiments with three individuals in each replicate. *UAP*: UDP-N-acetylglucosamine pyrophosphorylase gene, *CHSA*: chitin synthase gene A, *CHSB*: chitin synthase gene B.

In addition to the down-regulation of trehalase expression, an examination of an indirect up-regulation of trehalases by the injection of trehalose was conducted. The results showed that the trehalose injection increased the mRNA levels of *SeTre-1* and *SeTre-2* and significantly increased the expression of *UAP*, *CHSA* and *CHSB* at some time points (Figure 10). This further confirmed that the expression patterns of the genes in the chitin biosynthesis pathway are affected by changes in the expression of trehalase genes.

## Discussion

### Characterization of the two trehalase genes

In two lepidopteran insects, *Bombyx mori* and *Spodoptera frugiperda, Tre-1* and *Tre-2* were expressed in the midgut[14], [22], which is consistent with our findings (Figure 3). However, we found that the two trehalases in *S. exigua* were distributed in other tissues as well, including the cuticle, tracheae, Malpighian tubules and fat body. A possible explanation is that two trehalases are devoted to utilizing intracellular and extracellular trehalose to supply energy and material for the whole body, and the soluble trehalase is located in haemolymph that flows throughout the body. The expression patterns of SeTre-1 and SeTre-2 were different in the tissues (Figure 3). The high expression of SeTre-1 in the cuticle suggests its close relationship with slough during metamorphosis[23], while the high expression of SeTre-2 in the tracheae, the main structure of wings, may suggest its contribution to flight in the adult stage.

The expression of *SeTre-1* gene in the prepupal stage was found to be much higher than in other stages (Figure 4), which may be related to a greater requirement of chitin and energy during larva-pupa metamorphosis. A major peak of *SeTre-2* mRNA level was found in the mid-pupal stage, which may meet the high requirement for chitin synthesis and energy supply for tissue-reconstruction and pupa-adult metamorphosis. Additionally, the results of enzyme activity assay suggest that SeTre-1 plays a crucial role in the larva-pupa and pupa-adult stages, and SeTre-2 contributes more in the pupa-adult stage, which substantiated above assumptions.

### Specificity of RNAi for the two trehalase genes

The results of target gene mRNA levels in the present study were due to combined effects of RNAi action and feedback mechanism of the two trehalases. The specific down-regulation of the target gene transcript level within at least 24 hours post-injection was clearly observed (Figure 5C), which makes it possible to differentiate the functions of the two trehalase genes. Such specificity is in agreement with the gene-specific interference of several genes, such as the chitin synthase genes[18], chitinase-related genes[19], and nuclear receptor HR3 genes[20]. The decrease of *SeTre-2* after 24 hours in response to ds*SeTre-1* injection and the decrease of *SeTre-1* at 24 hours in response to ds*SeTre-2* injection, on the other hand, represents another mechanism of feedback or self-regulation other than non-specific RNAi (Figure 5C). As reported, the transcriptional level of *SeTre-2* is down-regulated followed by decreasing activity when the activity of SeTre-1 is inhibited and endogenous glucose is lacking; and *SeTre-1* mRNA level dropped when endogenous glucose is excess and trehalose synthesis is activated [24].

### Two trehalase genes have different functions in chitin biosynthesis

As reported in a coleopteran insect *T. castaneum*, the two chitin synthase genes and chitinase family genes have distinctly different functions[18], [19]. Here, we report the different functions of the two trehalase genes from a lepidopteran insect, *S. exigua*, in chitin biosynthesis for the first time.

First, the two trehalases use different resources of trehalose in insects. Treh-1 is a cytosolic enzyme involved in the hydrolysis of endogenous trehalose, while Treh-2 is an extracellular enzyme proposed to have a role in the assimilation of exogenous trehalose as a carbon source[25], [26]. After RNAi for *SeTre-1*, the expression level of *SeTre-2* dropped significantly in the prepupal stage, leading to a severe shortage of glucose that caused a pupation malformation (Figure 5C, Figure 8). This result demonstrates that the activity of SeTre-2 dropped when trehalose synthesis was stopped[24]. After RNAi of *SeTre-2,* however, the reducation of *SeTre-1* expression led to an abundance of trehalose and a shortage of glucose after 24 hours that caused a reduced body size(Figure 5C, Figure 8). In the puapl stage, there was sufficient glucose for the larva-pupa stage, but a severe shortage of trehalose that led to eclosion malformation (Figure 5C, Figure 8). In other words, SeTre-1 and SeTre-2 have different effects on the concentrations of glucose and trehalose, which implies that they may have different functions in insect chitin biosynthesis through the chitin biosynthesis pathway and the glycometabolism pathway.

Second, the interference of the *SeTre-1* and *SeTre-2* caused decrease in the survival rates at different times. The remarkable reduction of transcriptional levels of *SeTre-1* and *SeTre-2* at 72 hours post-injection caused by RNAi of *SeTre-1* resulted in high mortality during the larva-pupa stage, while the severe shortage of trehalose before pupal stage owing to RNAi of *SeTre-2* caused high mortality during the pupa-adult stage (Figure 6) and a high percentage of failure in eclosion (Figure 7). The phenotypes caused by RNAi are due to combined effects of RNAi action and feedback mechanism of the two trehalases as discussed above.

Eight enzymes are involved in the insect chitin biosynthesis pathway, starting with trehalase[5]. After the gene-specific interference of *SeTre-1* and *SeTre-2*, the *UAP* gene expression was affected only by the injection of dsRNA to *SeTre-2*, and the expression of the *CHSA* and *CHSB* genes were affected by RNAi for both *dsSeTre-1* and *dsSeTre-2* (Figure 9). The possible reason is that *CHSA* mainly exists in cuticle, the primary location for slough[27], and Tre-1 is regulated by 20E to participate in slough during the larva-pupa transformation[2]. On the other hand, Treh-2 was involved in incorporating trehalose from the blood into muscular cells and then providing the energy required for the visceral muscles to strongly support the peristaltic movement of the midgut during active feeding[23], *SeTre-2* appeared to be involved in food intake and the muscle movement of midgut, thus it may have a greater effect on *CHSB*. Besides downregulation of trehalase expression, the overexpress of trehalase genes by injection of trehalose increased the mRNA levels of key genes in the chitin biosynthesis pathway (Figure 10). These further confirmed that expressions of the genes in the chitin biosynthesis pathway are affected by changes in expressions of trehalase genes.

The two trehalase genes have different effects on *CHSA* and *CHSB*, which was supported by the chitin content measurements. The chitin content in the epidermis was mainly affected by *dsSeTre-1*, and that in midgut was only affected by *dsSeTre-2* (Table 2). Because CHSA is responsible for chitin synthesis in the cuticle and trachea and CHSB mainly for chitin synthesis in the peritrophic matrix in the gut[15], [18], it can be concluded that *SeTre-1* affected the expression of *CHSA* and chitin synthesis in the cuticle more significantly. *SeTre-2*, however, has a major effect on the expression of *CHSB* and chitin synthesis in the midgut.

### Possible mechanisms for two trehalase genes to affect chitin biosynthesis

**Figure 11:**
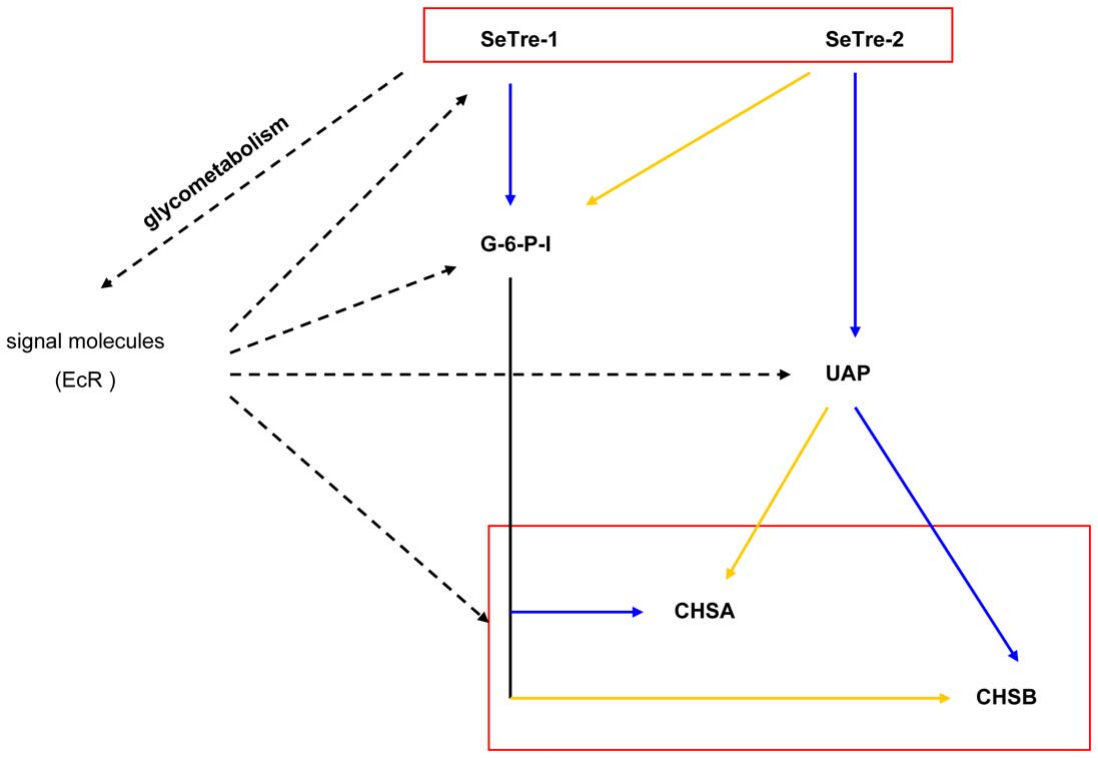
Possible mechanisms for the two trehalase genes to affect chitin biosynthesis. Solid arrows in the picture represent the internal interaction of the pathway genes, and broken arrows represent indirect effects through the glycometabolism pathway. The solid blue arrows represent greater effects, and the solid yellow arrows represent lower effects. *G-6-P-I*: glucose-6-phosphate isomerase gene, *UAP*: UDP-N-acetylglucosamine pyrophosphorylase gene, *CHSA*: chitin synthase gene A, *CHSB*: chitin synthase gene B.

The insect chitin biosynthesis pathway starts with trehalase. As reported, the enzyme activity, which can be affected by substrate feedback mechanisms, is controlled at both the levels of transcription and post-translation[27], [28], [29], [30]. In the present study, RNAi of the two trehalase genes decreased their transcription levels (Figure 5C), and their enzyme activities dropped accordingly (Figure 5E). In this way, chitin, the last product in the chitin biosynthesis pathway, is affected through changes in the substrates, products and activities of a series of enzymes in the pathway. On the other hand, the interference of the two trehalase genes resulted in a shortage of glucose (Figure 8) and decreased the transcription level of a signal molecule, ecdysone receptor (EcR) gene (data not shown). Moreover, the transcription levels of the pathway genes such as *SeTre-1*, *G-6-P-I*, *UAP*, *CHSA* and *CHSB* were affected at different times after RNAi of *SeEcR* gene (Yao, et al, unpublished data in the laboratory). In short, possible mechanisms for the two trehalase genes to affect chitin biosynthesis were summarized (Figure 11). Additional research will be conducted in the near future.

## Materials and Methods

### Insect cultures

*S. exigua* larvae were reared at 25±1°C with an L14:D10 photoperiod using an artificial diet [27], [30]. The developmental stages were synchronized at each molt by collecting new larvae or pupae. The midgut, fat body, cuticle and other tissues from the fifth instar larvae were dissected in insect saline containing 0.75% NaCl and stored at −80°C until further use.

### RNA isolation, cDNA synthesis and rapid amplification of the full-length cDNA

Total RNA was isolated at day two of the fifth instar larvae using TRIzol reagent (Invitrogen, USA) and the first strand cDNA synthesis was carried out according to the reverse transcriptase XL (AMV) (TaKaRa, Japan) protocol with oligo dT_18_. The first-strand cDNA (1 µL) was used as a template for PCR, and the components of the PCR mix were PCR buffer containing 0.1 mM dNTPs, 5 mM each primer, and 1.0 U of HiFi-Taq DNA polymerase (Transgene, China) in a total volume of 25 µL. Two pairs of degenerate primers (Table 3), *SeTre-1*-F1, *SeTre-1*-F2 and *SeTre-1*-R1, *SeTre-1*-R2 were designed from the conserved *SeTre-1* cDNA sequences of other insects. The first PCR reaction was performed with primers *SeTre-1*-F1 and *SeTre-1*-R1 using the following conditions: three cycles of 30 s at 95°C, 30 s at 45°C and 60 s at 72°C followed by 30 cycles of 30 s at 95°C, 30 s at 48°C and 60 s at 72°C. A second PCR was carried out using the nested primers *SeTre-1*-F2 and *SeTre-1*-R2 using the same conditions as for the first PCR. The amplified product was separated on an agarose gel and purified using the Gel Extraction Kit (OMEGA, USA). Purified DNA was ligated into the pMD18-T vector (TaKaRa, Japan) and sequenced completely in both directions.

**Table 3:** PCR primers used in this study.

Primers	Primer sequence
Degenerate primers	
*SeTre-1*-F1	5′-AGYGGYTGGGAYTTCTC-3′
*SeTre-1*-F2	5′-TGGATYATBGAAGGTCT-3′
*SeTre-1*-R1	5′-GCCADGCGTTRGGGAAGTCC-3′
*SeTre-1*-R2	5′-CGCRTCRTAYTTCTCRAACAT-3′
For cDNA cloning	
5-*SeTre-1*-1	5′-CACTATATTCGGATCGACAG-3′
5-*SeTre-1*-2	5′-GGAGCCAGGTTAGATGGGT-3′
3-*SeTre-1*-1	5′-CACACCAGATACATCATACC-3′
3-*SeTre-1*-2	5′-GGAATCACCGGGATGCTG-3′
For real-time PCR	
Q*SeTre-1*-F	5′-ATTCGCCAGAAACATCACCAAC-3′
*QSeTre-1*-R	5′-TTCCACTTATCAGCAGACCTCC-3′
*QSeTre-2*-F	5′-GGACTCTTGGGTTGATGGTGT-3′
* QSeTre-2*-R	5′-AGGCTTCTCAGTTCCGTGTAGG-3′
Q*Actin*-F	5′-TGCGTGACATCAAGGAGAAGC-3′
Q*Actin*-R	5′-CCATACCCAAGAAGGAAGGCT-3′
* QG-6-P-I*-F	
* eG-6-P-I*-R	
* QUAP*-F	5′-AGCAGACGGCAGACTAACTTTC-3′
* QUAP*-R	5′-GGACTCCTTCGTGGTCAACATAA-3′
* QCHSA*-F	5′-TAAGGCAAAGATTCGTCACAGG-3′
* QCHSA*-R	5′-CAGGGTCAGCAGATAGGTGTTC-3′
* QCHSB*-F	5′-CGCTGAGTCTTGTTGGTCCTGT-3′
* QCHSB*-R	5′-TCCACGCTACCTCTTTCCCTA-3′
For dsRNA synthesis	
ds*SeTre-1*-F	5′-ACCAGGAACCGTCAGTAG-3′
ds*SeTre-1*-R	5′-GCAACCATAGCTGTCAACA-3′
ds*SeTre-2*-F	5′-GCCAGGACAGGTTCACATC-3′
ds*SeTre-2*-R	5′- GCTTCACCATCGGAATTAGG-3′
*GFP*-F	5′-AAGGGCGAGGAGCTGTTCACCG-3′
*GFP*-R	5′-CAGCAGGACCATGTGATCGCGC-3′

F: forward, R: reverse.

A BD SMART RACE cDNA amplification kit (BD Bioscience Clontech, CA, USA) was used to obtain the full-length *Tre-1* cDNA. Specific primers, 5-*SeTre-1*-1 and 5-*SeTre-1*-2, for 5′-RACE and 3-*SeTre-1*-1 and 3-*SeTre-1*-2 for 3′-RACE (Table 3) were synthesized based on the cDNA sequence obtained from the identified fragment. PCR was performed with the *Tre-1*-1 primer and Universal Primer Mix (UPM, Clontech) by denaturing at 95°C for 30 s, 35 cycles of 95°C for 30 s, 55°C for 30 s and 72°C for 2 min, followed by a final extension at 72°C for 10 min. Nested PCR was carried out with the first- round PCR product as a template and the Nested Universal Primer A (NUP, Clontech) and *Tre-1*-2 primers. The RACE products were purified and sequenced as described above[16].

### Analysis of the cDNAs and protein sequences of *SeTre-1*

The sequences of the two trehalase cDNAs were compared with other trehalase sequences deposited in the GenBank using the “BLAST-N” or “BLAST-X” tools at the National Center for Biotechnology Information (NCBI) website. The amino acid sequence were deduced from the corresponding cDNA sequences using the translation tool at the ExPASy Proteomics website (http://expasy.org/tools/dna.html). Other protein sequence analysis tools used in this study, including MW, pI, and topology prediction tools, were obtained from the ExPASy Proteomics website (http://expasy.org/). Multiple sequence alignments of deduced amino acid sequences were made using the ClustalW multiple-alignment software (http://www.ebi.ac.uk/clustalw/index.html). The phylogenetic tree was constructed using the MEGA 3.1 software based on the amino acid sequences of the known trehalases. A bootstrap analysis was carried out and the robustness of each cluster was verified in 1000 replicates.

### Expression of recombinants and antibodies production for SeTre-1 and SeTre-2

Two cDNA fragments containing the *SeTre-1* partial sequence (547–927 bp) and *SeTre-2* partial sequence (1438–1863 bp) were inserted into the pET32a vector (Takara) for the expression of recombinant SeTre-1 and SeTre-2 proteins (rSeTre-1 and rSeTre-2) according to the manufacturer's instructions. After purification with an Ni Sepharose™ 6 Fast Flow (GE Healthcare), rSeTre-1 and rSeTre-2 were used to immunize rabbits as described previously[31]. The sera of the immunized rabbits were collected as the anti-SeTre-1 and anti-SeTre-2 sera.

### Western-blotting analysis for SeTre-1 and SeTre-2

Individuals or tissues were homogenized in two volumes of cold PBS (20 mM sodium phosphate and 130 mM NaCl, pH 7.2) plus protease inhibitor cocktail. The homogenate was centrifuged at 12,000×g for 20 min at 4°C. The supernatant was then incubated at 70°C for 10 min to remove impurity, the homogenate was centrifuged at 12,000×g for 10 min at 4°C again. The supernatant was immediately mixed with an equal volume of buffer for SDS-polyacrylamide gel electrophoresis and boiled for 10 min. After centrifugation at 12,000×g for 5 min, each supernatant was collected and stored at −40°C [32]. The amount of total protein in 1 ul sample buffer supernatant was determined by BCA kit. Electrophoresis on 12% SDS- polyacrylamide gels and Western-blotting analysis were carried out according to the methods of Mitsumasu et al. (2008). A purified primary antibody (anti-SeTre-1 or anti-SeTre-2 antibody, 1∶1000 dilution) and a secondary antibody (1∶5000 dilution), an anti-rabbit lgG antibody conjugated with HRP (BOSTER), were used.

### Immunocytochemical analysis of SeTre-1 and SeTre-2 in various tissues

Small pieces of midgut and cuticle were prepared from the fifth-instar larvae using a Frigocut cryotome (Mod. 2700, Reichert and Jung) at −26°C. The immunolabeling of the cuticle and midgut cryosections was performed according to Klein et al (1991).[33] The distributions of SeTre-1 and SeTre-2 immunoreactivity in the fatbody, tracheae and Malpighian tubule were investigated using the whole mount immunocytochemistry described previously[34]. For the localizations of SeTre-1 and SeTre-2, the sections and tissues were treated with a 1∶1000 dilution of anti-Tre serum. The control samples were treated with the preimmune serum. The visualization of the primary antibody was performed with HRP-conjugated anti-rabbit IgG (BOSTER). The sections and tissues were rinsed three times with PBS, covered with glycerol and viewed under a microscope.

### Developmental expression analysis of *SeTre-1* and *SeTre-2*

Total RNA was isolated from *S. exigua* on every day of its life cycle, including the larval, pupal and adult stages. 10 ug total RNA extracted from each stage was used as template to demonstrate the stability of *Seβ-actin*. The PCR reaction was performed with primers Q*Actin*-F and Q*Actin*-R using the following conditions: 95°C for 30 s, 28 cycles of 95°C for 30 s, 60°C for 30 s and 72°C for 20 s, followed by a final extension at 72°C for 10 min. The expression of *SeTre-1* and *SeTre-2* were estimated by real-time quantitative PCR (qRT-PCR) using a LightCycler480 system (Roche, Germany) and SYBR Premix Ex Taq (Takara, Japan). Two pairs of primers, Q*SeTre-1*-F and Q*SeTre-1*-R and Q*SeTre-2*-F and Q*SeTre-2*-R (Table 3), were designed to determine the expression of *SeTre-1* and *SeTre-2*. The cycling for each reaction was done in a final volume of 10 µL containing 0.3 µL of the cDNA sample (or standard), 0.2 µL (l0 µmol/ml) of each primer, and 5 µL of SYBR premix Ex Taq. After 10 s of initial denaturation at 95°C, the cycling protocol consisted of 45 cycles of denaturation at 95°C for 5 s, annealing at 58°C for 15 s, and elongation at 72°C for 20 s. The *β-actin* (EU179846) cDNA fragment was amplified with the Q*Actin*-F and Q*Actin*-R primers (Table 3) as an internal control. Standard curves were obtained using a ten-fold serial dilution of pooled total RNA. All the data were presented as the relative mRNA expression (mean ± SEM).

### Injection bioassays and sampling

Early day one of the fifth instar larvae were used for the injection experiment because larvae at earlier stages proved to be too small for satisfactory injections (data not shown). All of the reagents and enzymes used for the dsRNA synthesis were from the T7 RiboMAX™ Express RNAi System Kit (Promega) and all primers are shown in table 3. Five micrograms of dsRNA were injected into the side of the abdomen of the larvae using a 10 µl micro-syringe (Hamilton), and the injection point was sealed immediately with wax as described previously[27]. Two controls were performed, an equivalent volume of ds*GFP* (all experiments) and no treatment (the survival rate analysis and pathway gene detection only). In the phenotypic observation experiment, each group had 40 individual larvae with three replicates, and the observation was performed every 12 hours after injection during the larva stage and every 24 hours during the pupal stage. In the target gene detection experiment, each group had 80 individuals with three replicates, and 10 larvae were selected randomly at 12 h, 24 h, 48 h and 72 h after the injection for mRNA level detection independently. An individual with more than a 10% of decrease of the target gene expression was regarded as an effective RNAi, which was used to calculate the efficiency of RNAi. In the pathway gene detection experiment, three individuals confirmed as effective above were used for the detection independently. In the chitin content assay, each group had 50 individuals with three replicates, and 30 abnormal larvae were chosen for experiment. In the trehalose and glucose concentration assay, each group had 30 individuals with three replicates, and 5 abnormal larvae mixed at 24 h, 48 h and 72 h after injection were chosen for experiment.

### Trehalase activity assay

Ten crude larvae, prepupae and pupae in the late part of the fourth instar stage, the prepupal stage and day seven of the pupal stage were used for the soluble and membrane-bound trehalase activity analysis of individuals for three independent experiments. Ten crude individuals and the haemolymph of ten individuals from each of the three stages above were used for the activity analysis of the two trehalases. In the target gene experiment, three effective individuals were selected randomly at 48 h and 72 h post-injection for activity measurement independently in three replicates. The assay of trehalase activity was carried out according to the method described by Tatun et al.[2], [3]

### Trehalose and glucose content assay by HPLC

The concentrations of trehalose and glucose in *S. exigua* larvae were determined using high performance liquid chromatography (HPLC) with a 2414 refractive index detector[35]. An acetonitrile-water mixture (75∶25) was used as the mobile phase on a Carbohydrate Column (4.6 mm×250 mm, Waters). The working conditions were as follows: the flow rate was 1 ml/min, and the detector temperature was 37°C. Before the quantitative and qualitative determination of the concentrations of the sugars in the samples, standard solutions of trehalose and glucose (Sigma) were prepared and run on the same column to obtain standard curves for each sugar. The concentrations of trehalose and glucose in the samples were then calculated using the standard curves above.

### Chitin analysis

Ten abnormal larvae cuticles and thirty abnormal larvae midguts were choosen for the chitin content detection for three independent experiments. A piece of cuticle or three midgut mixed segments were measured in one sample. The assay of the chitin content was carried out according to the method described by Arakane et al (2005).[18]

### Trehalose injection assay

Early day one of the fifth instar larvae were used for the injection of 500 ug of trehalose per individual (the fifith instar larva has about 250 ug trehalose in the haemolymph). Each group had 30 individuals with three replicates, and three larvae were selected randomly per time point for mRNA level detection by quantitative real-time PCR independently. The methods used were the same as above.
